# Determination of polycyclic aromatic hydrocarbons extracted from lichens by gas chromatography–mass spectrometry

**DOI:** 10.1016/j.mex.2022.101836

**Published:** 2022-08-28

**Authors:** Lina María Ortega Fernández, Diana Marcela Uribe Ante, Marco Tadeu Grassi, Rafael Garrett Dolatto, Nazly Efredis Sánchez

**Affiliations:** a Universidad del Cauca, Programa de Ingeniería Ambiental, carrera 2 #15N, Popayán, Cauca, Colombia; b Universidade Federal do Paraná, Jardim das Américas, Caixa Postal 19032, CEP 81531-980, Curitiba, Paraná, Brasil; c Universidad del Cauca, Departamento de Ingeniería Ambiental y Sanitaria, Facultad de Ingeniería Civil, carrera 2 #15N, Popayán, Cauca, Colombia

**Keywords:** PAHs, Lichen, Road traffic emission, Biomonitors, Traffic emissions, Road characteristics

## Abstract

Lichens are well-known biomonitors for semi-volatile pollutants, due to their ability to absorb and retain different chemical compounds such as Polycyclic Aromatic Hydrocarbons (PAHs), directly linked to levels in the atmosphere. Based on that, this paper proposes an analytical method capable of quantifying 16 EPA-PAHs from lichens found in an intertropical zone, as a natural alternative to typical capture methods, with the aim of monitoring atmospheres polluted by toxic compounds. An analytical protocol, including sample pre-treatment, followed by ultrasound extraction, clean-up in a chromatographic column, concentration and quantification by Gas Chromatography-Mass Spectrometry (GC-MS) using Selective Ion Monitoring has been developed. Additionally, a set of guidelines on lichen collection and sample handling is given, in order to achieve representative samples.

• Limits of quantification (LOQ) and detection (LOD) varied from 2.0 to 16 µg/L and 1.0 to 5.0 µg/L, respectively. Calibration curves had correlation coefficients higher than 0.99 in all cases.
• Validation of the method for determining PAHs concentration associated to 30 lichen samples collected along two roads, with high and low traffic volumes was carried out.
• The method showed good performance according to the sources of PAHs, traffic patterns and gradient in roads.

## Graphical abstract

**Figure:**
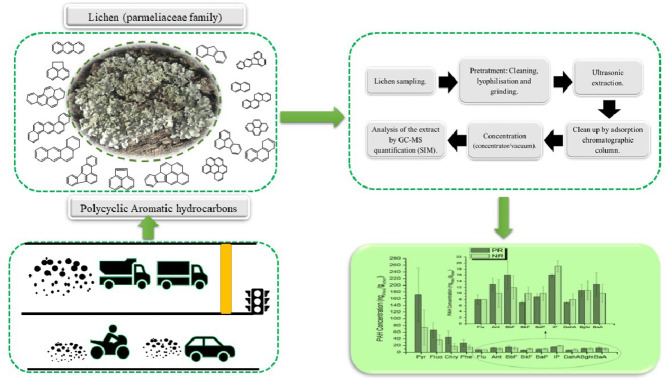

Specifications table

**Table:** 

Subject Area:	Chemistry
More specific subject area:	*Analytical chemistry*
Method name:	*Method for determining PAHs associated to lichens*
Name and reference of original method:	*N.A.*
Resource availability:	*N.A.*

## *Method details

The analytical methods used for EPA-PAHs quantification in lichens include sampling, pre-treatment (cleaning, drying by lyophilisation and grinding), ultrasound-assisted extraction, clean-up by adsorption in a chromatographic column, concentration by a rotational-vacuum concentrator and quantification by GC-MS in Selective Ion Monitoring (SIM).

## Sampling of lichen

The proper definition of a suitable collection method in a sampling campaign guarantees representative samples, avoids cross contamination, and minimizes the loss of analytes. We recommend the following steps for the appropriate collection of lichen samples for monitoring air quality that were considered in the present work.

In general terms, enough lichen samples (depending on pollution source exposure, kind of lichen, etc.) must be picked up from trees at minimum height of 1.6 m above the ground to avoid samples being contaminated during heavy rains, which could hinder the pre-treatment process. Forophytes must have a trunk diameter greater than 0.2 m, a trunk inclination less than 20° with respect to vertical axis and they should not to belong to excessively closed forest formations. Furthermore, morphological characteristics of trees must correspond to the healthy ones [1,2]. Lichens should not present evidence of human activities such as pesticide treatment or damage caused by animals. Another morphological aspect of lichen must be taken into account. These are, the general appearance of thallus, vitality, dryness, colour, and size, which could be an indicator of metabolic alteration following those described by Ederra, 1996 [3]. These recommendations could minimize uncertainty when this method is replicated, avoiding problems such as those mentioned by [4].

Tweezers, previously cleaned with acetone (HPLC grade) and electrically heated in an oven at 100 °C, were used to carefully separate the lichen thallus from the substrate. Field personnel wore gloves and disposable face masks. Samples were stored in aluminium bags in order to protect them from sunlight, avoiding PAHs photochemical degradation and preventing excess of moisture. The bags were labelled with a sample code and additional information was filled in a field form, including the date, site of collection, name of the the person who collected the sample, the number of tree (if any), the sample code in the aluminium bag and additional observations with qualitative information such as meteorological data.

Lichen samples were analysed immediately after their capture for determination of repeatability and recovery of the analytical method.

## Pre-treatment of lichen samples

Tweezers were used to manually remove lichen impurities such as ground, sand, tree bark, moss, or insects that had become adhered to the lichen. Subsequently, samples were refrigerated at -28°C for around 5 h and afterward dried by using a freeze-drying process (liophyliser, Liobras, L101). Samples were subjected for 18 h to lyophilisation with initial conditions of temperature (T): -54 °C, voltage (V): 219 V, Pressure (P): 205 µm Hg and final conditions of T: -58 °C, V: 219 V, P: 62 µm Hg.

The lyophilisation dehydration method has been considered in order to minimize the loss of volatile analytes, especially those with low molecular weight. Finally, samples were crushed in a ceramic mortar into a fine powder and stored in non-permeable amber storage bottles with screw cap (Supelco, Ref. No. 23233) to prevent volatile analytes from escaping.

## Extraction of PAHs

0.4 g (dry weight) of powdered lichen with 40 µL of surrogate standard (p-terphenyl-d14, 48418, in methylene chloride, Supelco brand) (100 ng_p-terphenyl-d14_/g_lichen_) were placed in a conical glass tube. Subsequently, 2 mL of hexane: dichloromethane (3:2 v/v) (analytical grade) was added.

The sample, surrogate compound, and solvent were taken to an ultrasonic bath (Unique brand) for 10 min at room temperature and later placed in a Macro IV centrifuge separator (model EV: 025) operating for 5 min at 3000 rpm. Finally, 2 mL of extracted upper layer was taken in a 10 mL neutral glassware test tube with a screw cap.. The extraction procedure was performed three times for the same lichen pulverized, thus obtaining a combined extract of 6 mL for each sample. To obtain approximately 0.5 mL, the extract was concentrated in an rotary evaporator (Christ brand rotary evaporator, RVC model 2-18 CDplus) at 60°C for 20 minutes.

## Clean-up

Chromatographic columns were prepared with a stationary phase formed by glass wool, 2 g of activated silica gel, 1 g of activated alumina (activated at 130°C according to EPA 3630B) and 0.5 g of anhydrous sodium sulphate. 15 mL of hexane: dichloromethane (HPLC grade) in 3:2 (v/v) ratio as mobile phase was passed through the column in order to condition the solid phase, such as was done by Studabaker et al. (2017)[5]. Subsequently 0.5 mL of raw extract was loaded to the column. The solvent containing the analytes was eluted with mobile phase (15 mL) yielding a final extract of approximately 8 mL. These stationary and mobile phases were chosen after a test that will be shown later.

## Tests of clean-up and lichen weight

Optimization of clean-up experimental conditions was developed in two tests by using a univariate analysis methodology. On the one hand, SG-A (silica gel- alumina) and ASG-AA (activated silica gel- activated alumina) were tested in order to choose a better stationary phase. In each case, 15 mL of dichloromethane was used as a phase mobile. Additionally, anhydrous sodium sulphate and glass wool were used in each column. On the other hand, the second test was devoted to the evaluation of the proper solvent by testing dichloromethane and hexane-dichloromethane (3:2 v/v). The best stationary phase found in the first test was used. Additional steps of the analytical method for all tests were implemented as previously explained.

ASG-AA had better performance which can be explained due to the high polarity of this column packing, allowing to PAHs (with low polarity) to elute from the column. The best eluent solvent was a mixture of hexane-dichloromethane 3:2 (v/v). Conversely to stationary phase, these solvents with low polarity are related to PAHs, easily dragging them through the column. These findings coincide with those presented by Blasco et. al. (2007) [6].

The optimization of the lichen mass was carried out by testing two different quantities of powdered lichen masses (0.3 g and 0.4 g in dry weight). This test is important to define an ideal quantity for the samples so that contamination of chromatographic equipment is avoided, but it is still high enough to be detected by this method. According to concentration given by the GC-MS, 0.4 g showed the best results with the best characteristics in their chromatograms .

## Concentration

The 8 mL of eluted extract from the clean-up was evaporated at 60°C for 30 min. Such a temperature favours the total solvent evaporation while keeping the EPA-PAHs. Afterwards, the analyte was dissolved in 225 μL of dichloromethane and 25 μL of internal standard (100 ng_internal_standars_/mL) (Semivolatile Internal Std Mix, CRM5M07296, in dichloromethane). Table 1 specifies the corresponding internal standards for each16 PAHs.

**Table 1:** Internal standard used for each EPA-PAH.

Internal Standard	1PAHs
Naphthalene-d8	Naphthalene
Acenaphthene-d10	Acenaphthylene
	Acenaphthene
	Fluorene
Phenanthrene-d10	Phenanthrene
	Anthracene
	Fluoranthene
Chrysene-d12	Pyrene
	Benzo(a)anthracene
	Chrysene
Perylene-d12	Benzo(b)fluoranthene
	Benzo(k)fluoranthene
	Benzo(a)pyrene
	Indeno(1,2,3-cd)pyrene
	Dibenz(a,h)anthracene
	Benzo(g,h,i)perylene

1 PAHs: Polycyclic Aromatic Hydrocarbons.

## Chromatographic analysis

The chromatograph was equipped with a triple quadrupole mass spectrometer, Brand Shimadzu QP2010-TQ8040, and an autosampler combi-PAL Shimadzu AOC5000. Splitless (270°C, 1μL) was used as injection mode. The capillary column was a DB-5ms (Agilent), 30 m × 0.25 mm ID × 0.25 µm film thickness. The helium (99.999 % purity) was used as carrier gas at a flow of 1.20 mL/ min.

The chromatograph oven worked with a temperature program that started at 50°C held for 5 min, then raised at 5°C/min up to 230°C; a second heating rate of 2°C/min up to 250°C and finally a third heating rate of 5°C/min up to 300°C held for 8 min. The transfer line and ion source temperatures were set at 280°C and 230°C, respectively. The retention time for each analyte and their corresponding ion monitored can be found in Table 2.

**Table 2:** Retention time and monitoring ion profile.

Compounds	Retention time (min)	Monitored ions (m/z)
Naphthalene-d8	7.87	136, 137, 108
Naphthalene	7.91	128, 127, 129
Acenaphthylene	11.30	152, 151,150
Acenaphthene-d10	11.64	162, 164,160
Acenaphthene	11.71	153, 154, 152
Fluorene	12.92	166, 165, 164
Phenanthrene-d10	15.11	188, 187, 184
Phenanthrene	15.16	178, 176, 152
Anthracene	15.29	178, 176, 179
Fluoranthene	17.99	202, 200, 203
Pyrene	18.51	202, 200, 201
p-terphenyl-d14	19.05	244, 243, 245
Benzo[a]antracene	22.29	228, 226, 229
Chrysene-d12	22.35	240, 236, 241
Chrysene	22.43	228, 226, 229
Benzo(b)fluoranthene	26.78	252, 250, 253
Benzo(k)fluoranthene	26.92	252, 250, 253
Benzo(a)pyrene	28.35	252, 250, 253
Perylene-d12	28.68	264, 260, 265
Indeno(1,2,3-cd)pyrene	33.39	276, 277, 274
Dibenz(a,h)anthracene	33.56	278, 279, 276
Benzo(g,h,i)perylene	34.13	276, 277, 274

## Calibration curves

The calibration curves were done by preparing 7 solutions in triplicated with different EPA-PAH concentrations (10, 25, 50, 100, 250, 500, 1000 µg/L) (PAH Mix- CRM48905, Supelco), adding internal standard and dichloromethane as solvent. The calibration curves were obtained by calculating the ratio of signals between standard compound and their internal standard versus PAH concentration. The correlation coefficients (R^2^) were between 0.993 and 0.999 (Table 3), which shows a good fit in the linear phase studied (10-1000 µg/L, 10-500 µg/L or 25-500 µg/L).

**Table 3:** Analytical characteristics of method for EPA-PAH and surrogate p-terphenyl-d14.

1PAHs	2LOQ	3LOD	Linear range	4R^2^
	µg L^−1^			
Naphthalene	9	3	10-1000	0.994
Acenaphthylene	6	2	10-1000	0.999
Acenaphthene	9	3	10-1000	0.993
Fluorene	9	3	10-1000	0.993
Phenanthrene	3	1	10-1000	0.994
Anthracene	6	2	10-500	0.999
Fluoranthene	3	1	10-500	0.999
Pyrene	9	3	10-1000	0.992
Benzo(a)anthracene	15	5	25-500	1
Chrysene	9	3	10-500	0.999
p- terphenyl-d14	3	1	10-500	0.999
Benzo(b)fluoranthene	6	2	10-500	0.997
Benzo(k)fluoranthene	12	4	25-500	0.993
Benzo(a)pyrene	6	2	10-500	0.994
Indeno(1,2,3-cd)pyrene	9	3	10-500	0.999
Dibenz(a,h)anthracene	9	3	10-500	0.999
Benzo(g,h,i)perylene	9	3	10-500	0.999

1 PAHs: Polycyclic Aromatic Hydrocarbons

2 LOQ: Limit of quantification

3 LOD: Limit of detection

4 R^2^: Correlation coefficient

Limit of detection (LOD) and quantification (LOQ) were calculated by Eqs. (1) and (2), where IC is the slope of calibration curves and σ is the standard deviation determined by of the intercept with Y-axis of at least 3 calibration curves.

(1) $$LOD=3.3\cdot \frac{\sigma }{IC}$$

(2) $$LOQ=10\cdot \frac{\sigma }{IC}$$

The lowest calibration point for each PAH is equal or higher than the LOQ, thus within calibration interval, data can be confidently achieved [7]. These facts can be verified in Table 3.

## *Validation of the analytical method

The present analytical method and sampling procedure were used to determine EPA-PAHs associated with thirty lichen samples collected in April of 2019 from different trees close to two roads in Municipio de Popayán – Colombia. Popayán is the capital of the Department of Cauca, located in southwestern Colombia in the Andean Region, specifically between the Western and Central Mountain Range.

**Fig. 1:**
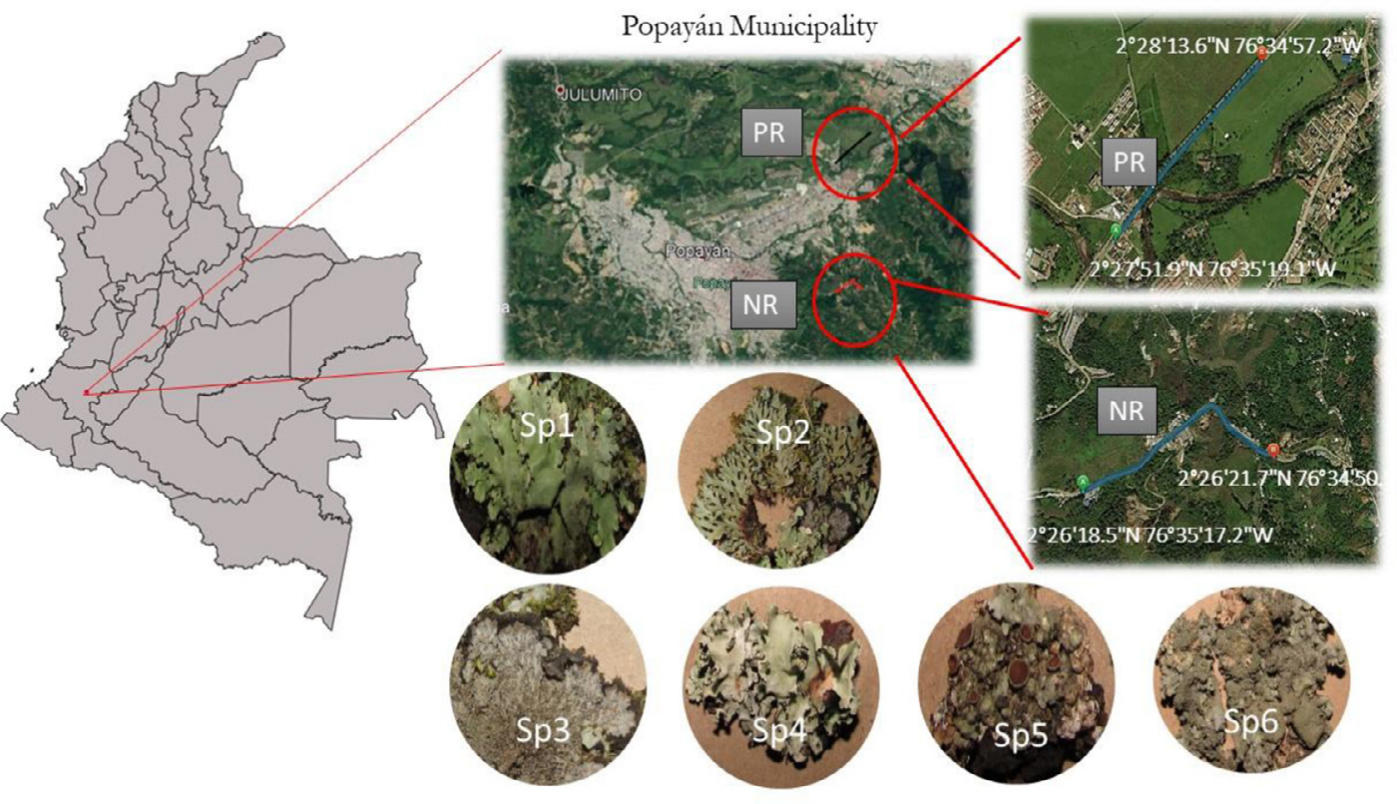
Location of sampling sites with coordinates in degrees, minutes and seconds (DMS) and photographs of lichen collected. Sp1: Hypotrachyna rhabdiformis (Kurok.) Hale; Sp2: Heterodermia obscurata (Nyl.) Trevis; Sp3: Parmeliella sp.; Sp4: Parmotrema perlatum; Sp5: Hypotrachyna sp.; Sp6: Punctelia colombiana Sérus. Species were deposited in the CAUP herbarium.

One sample set (n:15) was collected on the Panamericana Road (PR) that belongs to the Panamericana Highway, an important network of roads stretching across different American countries (between 2°27′51.9"N 76°35′19.1"W and 2°28′13.6"N 76°34′57.2"W). The second point was placed on the national route No. 20 (NR) (n:15), which serves as a connection between Popayan and the Department of Huila (between 2°26′17.4"N 76°35′19.5"W and 2°26′21.3"N 76°34′54.1"W). Fig. 1 shows the sampling sites as well as lichen collected. The species that were studied were deposited in the Universidad del Cauca herbarium (CAUP), which is a member of the Colombian Association of Herbariums (ACH). Lichen codes in herbarium are between 53069 and 53073.

Study roads were chosen because of their differences in types of vehicles, road capacity, traffic patterns, and the presence of speed control devices, in addition to providing easy access and safety to place field equipment. Such characteristics cause different fuel consumption and, thus, PAH emission levels associated with lichens. This factor was considered when evaluating the analytical method's performance in emissions from vehicular traffic based on road characteristics.

The identification of the lichen was carried out considering some specific characteristics belonging to the families Parmeliaceae, Physciaceae and Pannariaceae such as colour, lobe shape, and growth form.

The main characteristics of the studied road are shown in Table 4. PR has 20 times more motor vehicles than in NR, considering a global average, with a predominance of motorcycle, passenger cars, and vehicle with two axles. For its part, heavy duty tracks with three or more axles predominate in NR.

**Table 4:** Road segment studied and their main characteristics.

Road	General characteristics	Number of speed hump or speed bump^1^	Number of traffic lights	Approximate gradient for segment (%)
NR	Two-way vehicular road with two-lanes. Track with a bend.	0	0	2.6 to 12.8
PR	Two-way vehicular road with four-lanes. Linear track.	7 pairs of SSB, 2 BSH	1	< 3

1 SSB: small speed bumps, BSH: Big speed humps.

## Repeatability and recovery of the analytical method

Repeatability tests of the proposed method were carried out using samples of lichens from the families Parmeliaceae, Physciaceae and Pannariaceae collected in Federal University of Paraná, Brazil, following sampling procedure explained in present paper. Eight samples, each one of 0.4 g (dry weight) of lichen were analysed. Five samples of raw lichen were PAHs-enriched until obtaining a concentration of 100 ng_PAHs_std_ /g_lichen_ before the pre-treatment process. Lichen samples were enriched by applying the standard well distributed on the surface of the lichen using a transfer pipette. Another three samples were used as blank. Additional analytical steps such as pre-treatment (cleaning, drying, and grinding), extraction, clean-up, concentration and chromatographic analysis for each sample were carried out following the previously described procedures.

**Fig. 2:**
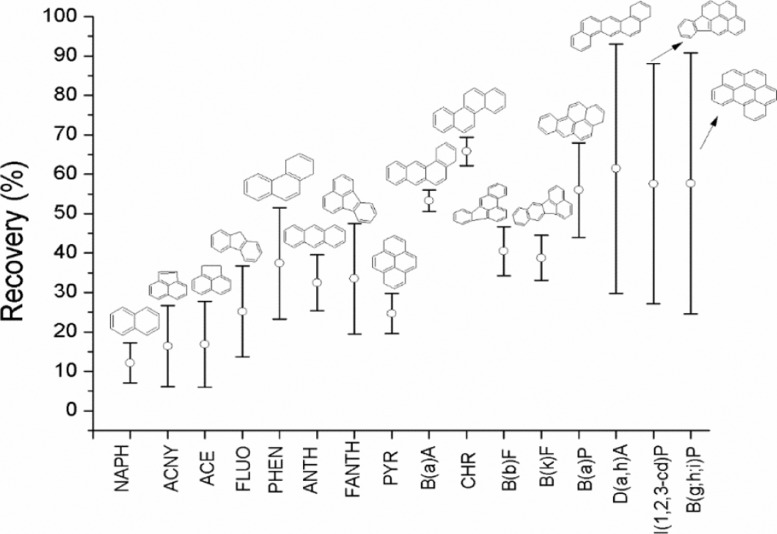
Recovery percentage (% REC) and confidence intervals for each PAH.

Fig. 2 shows the percentage of recovery (% REC) and confidence intervals (CI) calculated with 95% of confidence level (α: 0.05), for each PAH. Recovery from spiked samples was determined as described by Domeño et. al. (2006) [8] and % RSD by INMETRO (2003) [9].

Higher recovery percentages (% REC > 40 %) are associated with compounds with five and six aromatic rings, as well as B(a)A and CHR, which have higher persistent and lower volatility in the atmosphere than those with low molecular weight and higher vapour pressures. Furthermore, except for CHR, compounds with higher recoveries (D(a,h)A, I(1,2,3-c,d)P and B(g,h,i)P)have higher confidence intervals. B(a)A and CHR with the same molecular weight have very good repeatability and fair recoveries. Recoveries reported were used to correct the concentrations found in lichen samples.

Two issues can point to understanding the limited recovery rates in the present work. On the one hand, recovery rates may show that spiked 16 PAHs standards were possibly not fully absorbed by raw lichens; contrary to the case of the powdered lichen with a higher specific surface. This fact emerges when considering that the samples were exposed for a period of 24 h. On the other hand, the actual effect of the pre-treatment process on the added standard and in general on the PAHs present in the lichen samples could have effect on the loss of target compounds, mainly those of lower molecular weight, and particularly during the drying and grinding phases.

Concerning the first issue, Augusto et al. (2015) [10] reported that when lichens were exposed to FLU and B(a)P, the majority of B(a)P was absorbed after 8 days of exposure with accumulation in the lichens’ algal layer, allowing an understanding of the uptake lichen and ability to accumulate semi-volatile organic compounds. Accordingly, the lichen morphology presented resistance to both compounds and this could depend on their molecular weights. However, 8 days seems like a long time for the adsorption of aromatics in raw lichen, which can lead to analyte losses. This time should be a factor to consider in the performance analysis of similar methods in the future.

Although some other methods present higher recovery rates than the ones reported [6], the recovery levels in the present work were based on the fortification with the PAH standard spiked directly onto lichen prior to the pre-treatment process. This fact contributes to a complete understanding of method to predict values of PAHs concentration when the samples are collected from actual environments. Most methods using lichens as biomonitors published in literature, spike the EPA-PAH standard directly onto lichen pulverized after the pre-treatment process [6,11,5,12], which facilitates their retention by high specific surface and minimizes their volatilization when subjected to subsequent analytic processes. It is important to emphasise that this is true in analytical methods, including pre-treatment steps.

In this sense, sampling procedure, but mainly sample preparation (pre-treatment). could be a bottleneck in determining aromatic compounds associated with lichens. However, additional studies on method performance by comparing PAH enrichment in raw lichen and powdered lichen, should be carried out to confirm or discard this hypothesis.

To the best of our knowledge, little attention has been given to the pre-treatment processes of lichen samples, with a focus mainly on analytical or extraction techniques. This finding highlights that additional concerted effort toward understanding the effect of drying and grinding on loss of analyte in lichen samples should be made as it could affect recoveries presented in literature and used as an indication of the behaviour of the method in real samples. This fact together with that caused by extraction, concentration and clean up, could greatly affect the levels of recovered analytes.

Another issue associated with the limited recovery percentages in raw lichen can also be linked to the interference caused by the phyto-biological origin of the samples, e.g., the presence of chlorophyll in this matrix can cause suppression or increase of analytes in lichen samples.

Notwithstanding the previously mentioned and a complex biological matrix, the RSD ranged from 4.2% and 22.1% for the most cases, showing good repeatability.

## Lichen PAH concentration in PR and NR

A higher total PAH concentration in lichens was found in PR samples. Thirteen of sixteen PAHs were found in both study areas, with predominance of Fluo, Pyr, Chry and Phe. Aromatic compounds like Nap, Ace, and Acy were found to be below the LOD of the method, which corresponds to aromatics with 2 and 3 aromatic rings with higher volatility and lower persistence. The average total PAH concentration in PR was 374 ng_PAH_/g_lichen,_ meanwhile, NR corresponded to 152 ng_PAH_/g_lichen,_ with interval ranges varying from 16 ng_PAH_/g_lichen_ to 614 ng_PAH_/g_lichen_ and 62 ng_PAH_/g_lichen_ to 633 ng_PAH_/g_lichen_, respectively. This relation of 2.5 times higher in PR is explained by the concurrence of the vehicle fleet.

**Fig. 3:**
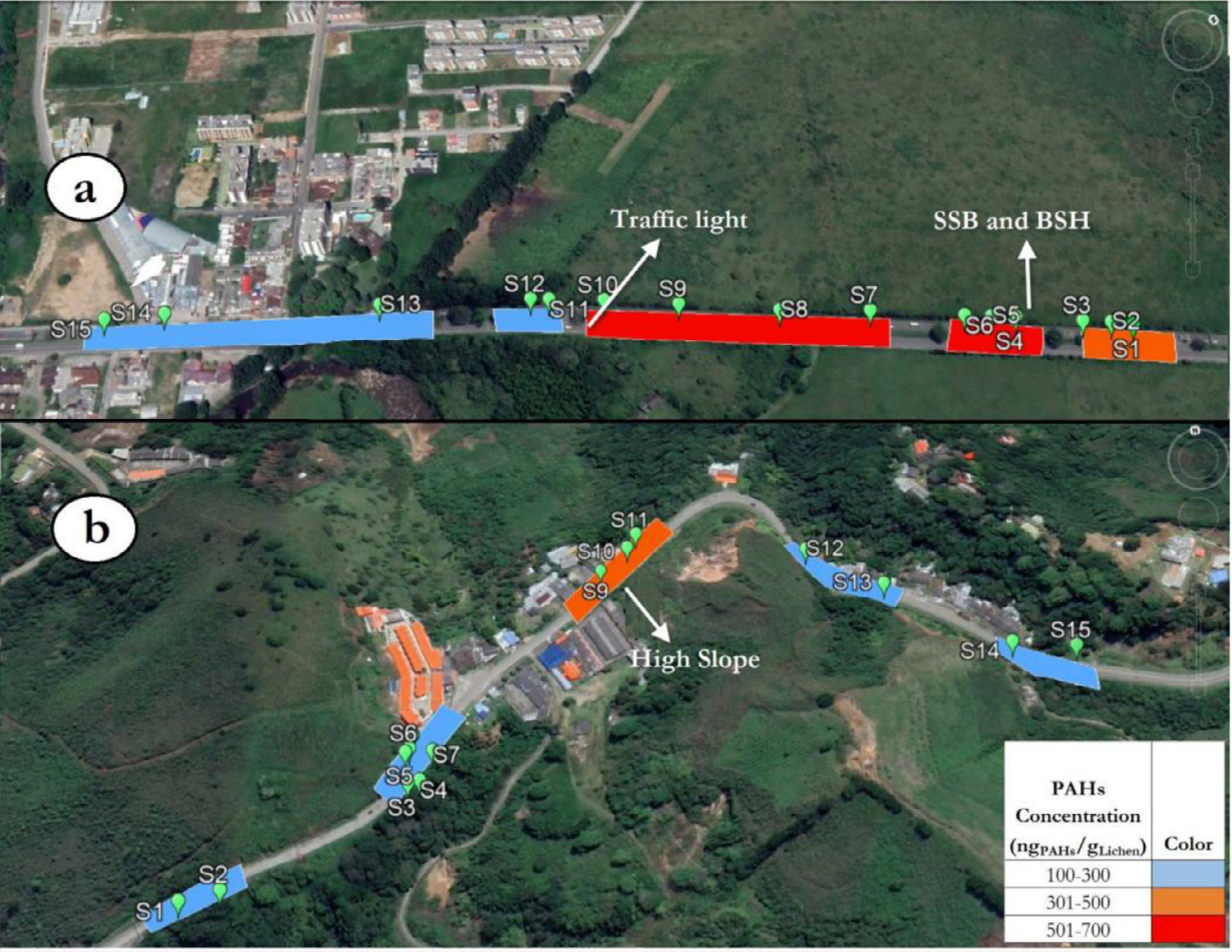
EPA-PAH sampling points and average concentration of in roads. a) PR and b) NR. Colors are directly related to average of PAH concentration found as associated to lichen in different transects. SSB: Small speed bumps, BSH: Big speed hump.

Fig. 3 shows the sampling points in PR and NR. The transects with colours correspond to concentrations of PAHs identified by the convention table. The sampling points were clustered (PR: S1-S3, S4-S6, S7-S10, S11-S12, S13-S15; NR: S1-S2, S3-S8, S9-S11, S12-S13, S14-S15) due to their proximity and similarity in traffic patterns and thus road characteristics. This strategy simplifies the analysis of results.

PAH concentrations in both roads provided by the present method are in accordance with traffic patterns and road specific features related to the level of fuel consumption, i.e. Higher PAH concentration is related to presence of traffic control devices (SSB, BSH and traffic lights) in PR and transect with high slope in NR. PR is the most polluted one due to its higher vehicular flow.

Considering trends in PR, high PAH concentrations associated to lichens are found in zone with the presence of 7 pairs of small speed bumps, 1 big speed hump, and 1 traffic light. Bumps, humps and traffic light force vehicles to reduce their speed. The subsequent fuel consumption in the acceleration phase increases pollution as reported by [13] and [14]. In absence of speed control devices, the most polluted zone in NR corresponds to road transect close to higher road gradient and a road bend. The higher emission of pollutant in sloped roads was previously reported by [15].

**Fig. 4:**
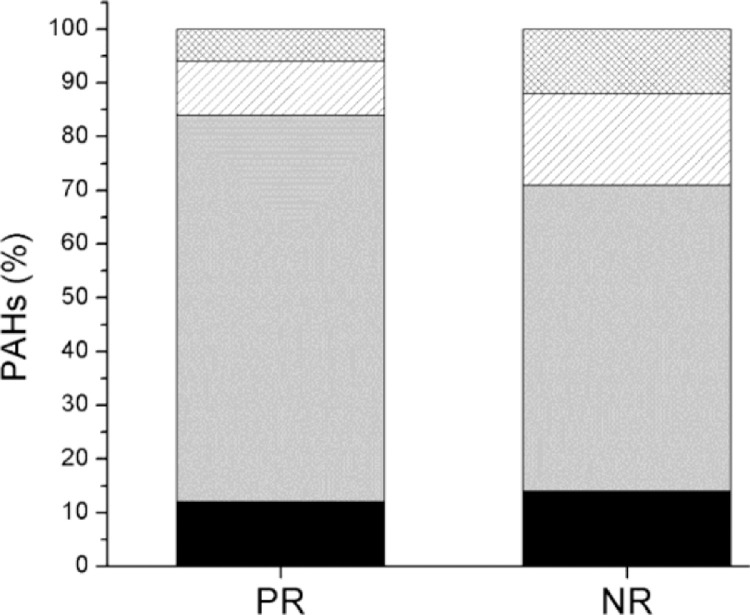
Distribution of EPA-PAHs according to their number of aromatic rings. The black box inside the bars shows analyte percentage with three aromatic rings, and thus grey to four aromatic rings, sloping lines to 5 aromatic rings, and grid box to six aromatic rings.

Fig. 4 shows the distribution of the percentage of PAHs according to their number of aromatic rings in both study points. A clear predominance of 5 and 6 aromatic rings is observed in NR, which was expected due to the prevalence of heavy fleet vehicles using diesel fuel (1.5 times more than in PR per week).

Under this line of principles, the method reported a good performance in reproducing the pollution generated on two roads according to their traffic patterns and road characteristics. This fact could lead the possibility of using this method to monitor, the effect of geometric designs and traffic dynamics on the generation of toxic pollutants at various sites at a lower cost. However, additional studies are required aiming to improve the present method and carry out a greater number of analyses on routes with different characteristics to confirm hypothesis here mentioned.

## *Additional information

PAHs are organic compounds with two or more fused aromatic rings of petrogenic or pyrogenic origin, derived from natural or anthropogenic processes. As a matter of fact, it is well known that some of these compounds have carcinogenic, mutagenic, teratogenic, and toxicological characteristics, that represent a high risk to human health [16]. Based on these properties, the EPA classified 16 PAHs as priority pollutants for environmental monitoring. This list is composed by naphthalene (NAPH), acenaphthylene (ACNY), acenaphthene (ACE), fluorene (FLUO), phenanthrene (PHEN), anthracene (ANTH), fluoranthene (FANTH), pyrene (PYR), benzo(a)anthracene (B(a)A), chrysene (CHR), benzo(b)fluoranthene (B(b)F), benzo(k)fluoranthene (B(k)F), benzo(a)pyrene (B(a)P), Indeno [1,2,3-cd] pyrene (I(1,2,3-c,d)P), dibenzo(a,h)anthracene (D(a,h)A), and benzo(g,h,i)perylene (B(g,h,i)P) [17]. EPA-PAH can appear in the particulate phase, associated with particulate matter, or in the gas phase.

Conventional monitoring of air compounds, such as particulate matter and consequently, PAHs, may be a difficult task in some scenarios due to the expensive nature of the air sampling equipment involved, as well as the physical accessibility to the sampling areas with these devices [8], all of which, combined with the fact of the high energy requirements for operation. This situation impacts more severely the monitoring activities, mainly in developing countries, where a higher risk to human health is caused by air pollutants [18]. A recent review study on PAHs levels in different atmospheric environments of cities in Latin America explains that scarce information is found in general for most countries [19]. Under this line of principles, the use of biomonitors provides an advantage for the assessment of the presence of pollutants, including organic compounds, such as PAHs. Among these biomonitors, lichens are known to be a biological monitoring tool for determining ecosystem health [5]. Lipophilic characteristics of the lichen surface attract compounds that share the same characteristics, such as PAHs, which facilitates their uptake [4].

Different studies have demonstrated efficiency of lichens as pollutant bioacumulators [5,20]; some of these works have also been devoted to lichens of the Parmeliaceae family [21].

According to the literature, analytical methods have been optimized for the quantification of PAHs in lichens, e.g. [6]. In general, they are carried out through a pre-treatment step, an extraction procedure, a clean-up scheme, and a method for the analysis of extracts. After the samples are collected, they are cleaned to avoid exogenous matter and thus interference in the analytical procedure. Lichens are usually subjected to drying. The authors mention various techniques for this purpose, such as drying out in an oven [12] or on a stove [22], or simply leaving the sample at room temperature [20]. Subsequently, lichens are ground in order to homogenize the samples by means of an agate mortar [12], a ball mill [20], or milled in liquid nitrogen [11].

Extraction procedures includes Soxhlet with high amount of chemical compounds used in the process [23], ultrasound-assisted extraction (UAE) [24], dynamic extraction with ultrasound-assisted solvent (DSASE), [25]; and more recently QuEChERs technique [12]. The strategies used for cleaning include solid phase extraction (SPE) [21] and column chromatography [5,11].

Chromatographic techniques for the determination of extracts containing PAHs include gas chromatography coupled to mass spectrometry or with time-of-flight mass spectrometry GC-MS/GC-TOF-MS [12,11], High Performance Liquid Chromatography (HPLC) [26] and Laser induced fluorescence (LIF) [27].

The studies on the efficiency of analytical methods for PAH determination from lichen have been focused mainly on the comparison of extraction techniques [12] or clean-up procedures [6]. For this purpose, the recovery percentage was used as a measure of method effectiveness, which was achieved through the enrichment of powdered lichen with the PAH standard.

The effectiveness of pre-treatment and even sampling processes has not received enough attention in the literature reviewed, particularly when both represent critical steps in the overall methods and their reliability, due to the sample handling and transformation involved. This fact could affect the analyte concentration found in lichens, contributing to errors.

In this context, a method for the quantification of PAHs from lichens is presented here, providing details from the pre-treatment process (cleaning, drying, and grinding) to the analytical procedure, which is not widely provided in literature. The proposed drying of samples consists of lyophilisation, a novelty to be used in this application, which allows the removal of water at low temperatures. This avoids the loss of analytes, mainly those with low molecular weight.

The UAE for extraction, clean-up in a chromatographic column, concentration and PAHs quantification by GC-MS were developed in this study. Additionally, optimization of the mobile and solid phase of chromatography column in the clean-up, as well as the weight of lichen, were carried out.

The method achieves its recovery rates through the enrichment of the samples with a standard solution containing 16 PAHs and the subsequent comparison with another non-enriched set (blank samples). Unlike several other methods reported in the literature, the enrichment process is applied directly to raw lichens, that is, prior to the pre-treatment process rather than powdered lichen. This fact contributes to the understanding of the capability of the overall method to obtain the values of PAH concentrations in the biological samples. In this regard, although the present work is a step forward, more efforts should be made to create additional strategies in such a way that the loss of target compounds can be minimized. This improvement may help to significantly diminish the loss of concentration of PAHs, particularly of those with lower molecular weight. Some factors, such as the adsorption standard time of PAH in raw lichens, the presence of chlorophyll, and the severity of pre-treatments processes, which could become a bottleneck, should be taken into account in future studies with the same focus.

Improvements in sampling procedures, as presented here, should also be considered to obtain representative lichen samples and to avoid losses or cross contamination of samples containing analytes of interest. This is particularly true when lichens are used for monitoring the presence of toxic compounds in the atmosphere of different places.

The PAH concentrations in samples of lichen collected on both roads by using the present method showed consistence with the source in each study area. There was discovered to be a relationship between aromatic levels and traffic intensity, type of motorized vehicle or fuels, traffic patterns and road characteristics. In this way, this method could be used to compare the prevalence or trends of PAH emissions when monitoring equipment is not available or when difficult-to-access areas are required to be monitored, a common issue in developing countries.

Finally, the method proposed for PAHs determination from lichens follows the principles of green chemistry, namely waste prevention and minimizing the total amount of solvent and auxiliary substances used. As a result, it is useful information for researchers looking for alternative methodologies that reduce solvent, electricity, and time consumption.

Table 5 shows experimental conditions for different kinds of extraction for the present method and those reported in the literature. The extraction step of aromatics from biomonitors is usually the most solvent- and time-consuming step in analytical methods. In this sense, these two factors are compared in the present section. The extraction of the present work consumes about of 60 to 90% less solvent than other methods using the same technique, with a reduction of between 0.5 and 1.5 h in time. Concerning other traditional techniques, such as soxhlet extraction, the reduction is more significant. DSASE is a little-used technique but may have potential to be used in future methods as it is geared towards green chemistry.

**Table 5:** Experimental conditions for the extraction procedure.

Extraction technique	Solvent volume (mL)	Extraction time (h)	References
Soxhlet	100	16	[28]
Soxhlet	300	24	[29]
Soxhlet	200	24	[26]
Dynamic sonication-assisted extraction (DSASE)	2 mL	0.17	[6]
Accelerated solvent extraction (ASE)	60	Not reported	[30]
Ultrasonic bath	15	2	[31]
Ultrasonic bath	60	1	[32]
Ultrasonic bath	60	1	[24]
Ultrasonic bath	6	0.5	Present work
